# Convalescent Diet

**Published:** 1912-07-20

**Authors:** 


### INSTITUTIONAL HOUSEKEEPING.

### Convalescent Diet.

Half the problem of feeding convalescents and sick people is to induce them to take their food. Therefore the housekeeper needs to be very particular in serving patients' diets. Doctors will usually point out particular foods, but they must leave it to the matron or nurse to see that they are cooked and served palatably. In most cases the digestive organs are impaired, therefore the food must be easy of assimilation, yet sufficiently nutritious to strengthen and help to restore the patient to health.

The chief rules to observe are the following:—

1. All food should be of the best quality and perfectly fresh.

2. The meals for the day should be prepared without the patient's knowledge, away from the sick-room, and as far as possible the smell of cooking should be excluded from the invalid's room.

3. All utensils used should be scrupulously clean as a patient's palate is particularly sensitive to carelessly prepared food.

4. The food prepared should be small in quantity; usually it is better to prepare just enough for one meal, the appearance of a large portion is sufficient to take away an already small appetite. As a patient soon tires of one dish, it is safer to prepare a little at a time.

5. Variety of food is of great importance, but doctor's orders must always be observed in choosing the meal.

6. Food must be selected that will be easily digested, and which possesses the requisite food value in a small bulk, as the appetite is usually slender.

7. No indigestible method of cooking, such as frying, should be employed. Steaming is the best method to use, as the full flavours are retained in the article cooked.

8. Invalid's food should be thoroughly and carefully cooked; it should be delicately seasoned and flavoured. No rich sauces should be included, and pepper being an irritant should be omitted.

9. In serving the meal a very dainty tray should be prepared with a spotless cloth, fresh flowers, shining silver, and sparkling glass. Great care should be exercised in carrying any food to a patient, so that none is spilt either on the cloth or in the saucer, as nothing is more likely to turn him against the meal if badly served.

10. No food left by the patient should remain in the sick-room, and in the case of infectious illness it should be burnt.

11. Something suitable should always be in readiness for an invalid, such as beef-tea, which properly prepared contains good nourishment. Barley water or weak lemonade should be kept at hand to quench thirst, especially at night. Many persons think jelly an ideal food for an invalid, but it contains no nourishment, and a person fed on it would soon lose strength. It is useful as a cooling sweet.

As a general rule patients that can take eggs and egg dishes, milk, and white fish are easy to feed. Not many invalids are allowed much bread or potato, on account of starch being difficult of digestion. The stalks of green vegetables are not given for the same reason.

Well made beef-tea, soups made from good material, and perfectly free from fat, are very useful in the diet. There are many light puddings made with egg which are easily digested, from the plain steamed custard to souffle mixtures, but any rich mixture should be avoided.

It is instructive to look through the above and ask whether persons in good health do not likewise require fresh food of good quality, bright glass and clean table-linen; whether they are not nauseated by the smell of cooking beforehand; whether they are not entitled to precision in the service and to easily digestible food. True, the appetite in healthy women will triumph over obstacles. But the ideals of the housekeeper in feeding the convalescent and the staff are less dissimilar than is often supposed.

### Housekeeping Queries.

Convalescent Diet.

All recipes suitable for convalescent patients could also be used for suppers for the nursing staff, as they are necessarily light and digestible. *Fish Creams* are very simple and inexpensive. Prepare as follows: Well grease some small cups or moulds. Remove sufficient raw fish from the bones and skin to weigh 4 oz.—fresh haddock answers well. Put the bones into a pan with cold water to cover and a little salt, simmer for fifteen minutes, then strain off and save the liquid. Melt ½ oz. of butter in a saucepan, add ½ oz. of fresh breadcrumbs and half a teacupful of milk. Bring to boiling-point, add the fish and one raw yolk of egg. Pound lightly, and rub the mixture through a wire sieve. Season it carefully and, if possible, add half a gill of cream or, if preferred, milk. Beat the white of egg to a stiff froth, stir it lightly into the mixture, and three-parts fill the tins with the latter. Cover with greased paper and steam gently for half an hour. Turn out and pour over a white sauce made from the fish stock.

Try *Marrow with Egg Sauce* for the vegetable course. Potatoes are at present waxy, and some patients cannot digest even the tenderest of peas. Note that the value of this dish is in the sauce; by its use the yolks of two eggs are administered in quite an unsuspected form. Obtain a small marrow, do not cut or peel it, but put it in the steamer and steam for about an hour or until tender: the time depends on the age and variety of marrow and the steady supply of steam. When cooked, peel the marrow and remove seeds after cutting it in quarters. In this way the marrow has not deteriorated through the action of water on cut surfaces. Lay neat pieces of marrow on a hot dish and coat with the following sauce: Melt 1 oz. of butter in a saucepan, mix smoothly with it barely ½ oz. of flour, add half a pint of milk, stir until it well boils. Add seasoning and the yolks of two raw eggs beaten up with a tablespoonful of milk. Reheat without reboiling, add a teaspoonful of chopped parsley, and use.

NOTICE TO MATRONS.

We are always pleased to answer queries and give recipes which may be asked for by our readers. Let us know if any difficulties are hampering you, and we will do our best to find a remedy. Any successful experiments you may be making in culinary matters would interest a wide circle.

